# The Edinburgh Postnatal Depression Scale: translation and validation for a Greek sample

**DOI:** 10.1186/1471-2458-9-329

**Published:** 2009-09-09

**Authors:** Victoria G Vivilaki, Vassilis Dafermos, Manolis Kogevinas, Panos Bitsios, Christos Lionis

**Affiliations:** 1 Department of Social Medicine, Faculty of Medicine, University of Crete, Heraklion, Greece; 2 Department of Midwifery, TEI, Athens, Greece; 3 Department of Political Sciences, University of Crete, Rethymno, Greece; 4 Centre for Research in Environmental Epidemiology, Barcelona, Spain; 5 Department of Psychiatry and Behavioural Sciences, University of Crete, Heraklion, Greece; 6 Clinic of Social and Family Medicine, Faculty of Medicine, University of Crete, Heraklion, Greece

## Abstract

**Background:**

Edinburgh Postnatal Depression Scale (EPDS) is an important screening instrument that is used routinely with mothers during the postpartum period for early identification of postnatal depression. The purpose of this study was to validate the Greek version of EPDS along with sensitivity, specificity and predictive values.

**Methods:**

120 mothers within 12 weeks postpartum were recruited from the perinatal care registers of the Maternity Departments of 4 Hospitals of Heraklion municipality, Greece. EPDS and Beck Depression Inventory-II (BDI-II) surveys were administered in random order to the mothers. Each mother was diagnosed with depression according to the validated Greek version of BDI-II. The psychometric measurements that were performed included: two independent samples t-tests, One-way analysis of variance (ANOVA), reliability coefficients, Explanatory factor analysis using a Varimax rotation and Principal Components Method. Confirmatory analysis -known as structural equation modelling- of principal components was conducted by LISREL (Linear Structural Relations). A receiver operating characteristic (ROC) analysis was carried out to evaluate the global functioning of the scale.

**Results:**

8 (6.7%) of the mothers were diagnosed with major postnatal depression, 14 (11.7%) with moderate and 38 (31.7%) with mild depression on the basis of BDI-II scores. The internal consistency of the EPDS Greek version -using Chronbach's alpha coefficient- was found 0.804 and that of Guttman split-half coefficient 0.742. Our findings confirm the multidimensionality of EPDS, demonstrating a two-factor structure which contained subscales reflecting depressive symptoms and anxiety. The Confirmatory Factor analysis demonstrated that the two factor model offered a very good fit to our data. The area under ROC curve AUC was found 0.7470 and the logistic estimate for the threshold score of 8/9 fitted the model sensitivity at 76.7% and model specificity at 68.3%.

**Conclusion:**

Our data confirm the validity of the Greek version of the EPDS in identifying postnatal depression. The Greek EPDS scale could be used as a useful instrument in both clinical practice and research.

## Background

The incidence of postpartum depression affects between 10% and 20% of new mothers and the clinical symptoms can appear as early as in the first weeks following delivery. However, postpartum depression often goes unrecognized with several consequences for the mother and the newborn [1-3].

The Edinburgh Post Natal Depression Scale (EPDS) has been specifically developed in order to screen for postnatal depression [4]. The EPDS is a sensitive screening instrument for the early detection of depressive symptoms as well as a sensitive instrument according to diagnostic criteria for major depression [5]. Use of the Beck Depression Inventory (BDI) [6,7] and BDI-II with postpartum samples has been reported in the literature as well correlated with EPDS [3,5] and other instruments used to screen for postnatal depression like Postpartum Depression Screening Scale (PDSS) [3].

With a cut-off score of 12/13 for screening English population it was reported sensitivity 86%, specificity 78%, Positive Predictive Value (PPV) 73% and alpha coefficient = 0.87 [4]. Although EPDS has been developed for English speaking populations, it has been translated and validated for non English speaking populations. However, not all validation studies include estimation of the cut-off scores that might be appropriate in different languages.

It has been observed through many validation studies that there is cultural variation in the expression of depressive symptoms during the postnatal period [8-10] that may result in differences in the psychometric characteristics of the EPDS [5,8,10] and differences in screening procedures.

A recent study reported that Postnatal Depression in a Greek urban area had an overall prevalence of 19.8% and a point prevalence of 12.5% at the end of the first month after delivery [11]. However, the actual rates of Postnatal Depression may be higher in that group, as the women were interviewed by phone and therefore may be reporting fewer symptoms [12]. Research has highlighted the wide impact of perinatal mental health problems and the public health role of community midwives in detection and initial assessment of perinatal mental disorder [13-15]. Since the profound effect of untreated postnatal depression is well documented [1-3,5,6], in clinical settings, identification of postnatal depression can be improved by increasing awareness and skills of health professionals in screening through the use of specific questionnaires, like EPDS. More specifically, efforts have been undertaken in Greece in screening by community health professionals in order to meet the women's health needs, as a potential benchmark of establishing an effective primary care system [16].

The general aim of this study was to translate and validate this instrument into Greek. More specifically the study's objectives were to:

1. Test a Greek version of the EPDS and assess its reliability and validity in identifying postpartum depression in a sample of new mothers.

2. Examine the factor structure of Greek EPDS.

3. Evaluate the sensitivity, specificity and predictive values of Greek EPDS over a range of cut-off scores.

## Methods

### Procedures

#### Greek version of EPDS - Translation and pilot study

The 10 items of EPDS were translated by two independent bilingual translators. One other native English speaker who did not have knowledge of the original instrument then back translated the re-conciliated Greek version. The backward translation was sent to a group of English experts for comments (health professionals with specialization in perinatal psychology). The translated questionnaire was culturally adapted through a cognitive debriefing process that was used to identify any language problems and to assess the degree of respondents understanding of the item's content that was meant to be elicited [17]. During this stage the reconciled Greek version of the EPDS was pilot tested with 8 mothers who had been admitted to Obstetric Gynaecology Clinic of University Hospital of Heraklion. As part of the cultural adaptation process, in-depth interviews were implemented about the respondents understanding of the questionnaire with the purpose of revealing inappropriately interpreted items and translation alternatives. The participants gave their impression on the clarity of the each item, the relevance of the content to their situation, the comprehensiveness of the instructions and their ability to complete it on their own. They were also encouraged to make suggestions whenever necessary. Finally, written comments made by the participants in the Cognitive Debriefing Report were included in the final Greek version of EPDS that was validated with the women who participated in the study.

#### Data collection

This study is part of a major project for translation and validation of screening instruments into the Greek language. After receiving ethical approval from the University of Crete, validation activities were initiated from June 2007 until February 2008. Following previous correspondence by mail and subsequent written informed consent, the mothers completed the EPDS and BDI-II questionnaires in the presence of a midwife (VV) at their homes or during their stay at the postnatal ward. The order of completion of the two questionnaires was counterbalanced; BDI-II was used in order to quantify the severity of any depressive symptoms. Along with the questionnaires there was a cover letter explaining the purpose of the study, providing the researchers' affiliation and contact information, and clearly stating that answers would be confidential and anonymity would be guaranteed in the final data reports.

#### Participants

130 women were recruited from the perinatal care registers of the Maternity Departments of 4 Hospitals of Heraklion municipality (2 public and 2 private). Inclusion criteria were fluency in spoken and written Greek language between 4 days till 16 weeks postpartum delivery of a live healthy infant and written informed consent. In total 120 mothers agreed to participate (rate of attendance 92.3%).

#### Instruments

##### Edinburgh Postnatal Depression Scale [4]

This is a 10-item self- report scale consisting of statements describing depressive symptoms. The 10 symptoms of depression included are: inability to laugh and look forward to things with enjoyment, blaming oneself unnecessarily, anxious or worried, scared or panicky, inability to cope, difficulty to sleep, sad or miserable, crying and thoughts of harming oneself. Each question has four possible answers, graded depending on the severity or duration of each symptom.

##### Beck Depression Inventory-II [18]

The recent revision of the BDI was used [19]. The BDI-II is a 21- item self report scale to measure the presence and intensity of depressive symptoms. Each item is scored on a 4-point scale ranging from 0-3. In particular, in BDI-II the symptoms of weight loss, body image change, work difficulty, and somatic preoccupation were deleted and replaced by the four symptoms of agitation, worthlessness, concentration difficulty, and loss of energy.

### Data analysis

Descriptive characteristics (including means, standard deviations, frequencies and percentages) were calculated for the sociodemographic variables. The assumptions of normality, homogeneity and independent cases of the sample were checked. Two independent samples t-tests were carried out to compare the EPDS scores in the groups of depressed and not depressed women according to BDI-II. Women were divided into four groups: no depressive symptoms (0-9) and those with mild (10-15), moderate (16-23) and severe (>24) depression symptoms. One-way analysis of variance (ANOVA) was used to compare the mean depression symptom levels - according to BDI-II scores- between the four groups of women.

#### Reliability

Reliability coefficients as measured by Cronbach's alpha were calculated for the EPDS and BDI-II in order to assess reproducibility and consistency of the instrument; the internal consistency of the Greek EPDS was also tested using Guttman split-half coefficients.

#### Factor structure

The underlying dimensions of the scale were checked with an explanatory factor analysis using a Varimax rotation and Principal Components Method as a usual descriptive method for analyzing grouped data [20]. Factor analysis using principal component analysis with varimax rotation was carried out to determine the dimensional structure of EPDS using the following criteria: (a) eigenvalue >1 [21]; (b) variables should load > 0.50 on only one factor and on other factors less than 0.40; (c) the interpretation of the factor structure should be meaningful (d) Screeplot is accurate in the case that the means of Communalities are above 0.60 [22]. Computations were based on covariance matrix, as all variables were receiving values from the same measurement scale [23]; A Bartlett's test of sphericity with p < 0.05 and a Kaiser-Meyer-Olkin (KMO) measure of sampling adequacy of 0.6 were used in performing this factor analysis. A factor was considered as important if its eigenvalue exceeded 1.0 [21]. As factor analysis found two independent subscales, subsequent Cronbach's alpha were separately carried out for each subscale, highlighting how the items group together. Additionally, a Confirmatory analysis -also called structural equation modelling- of principal components was conducted by LISREL (Linear Structural Relations) to confirm the scale items principally load on to that factor and correlate weakly with other factors, to assess tests for significance of factor loadings and orthogonality of factors [20,22,24]; a model -based on a priori information of exploratory factor analysis- was built in order to specify latent factors, their component variables and the intercorrelations of the response variables; maximum likelihood LISREL estimates, t-values, error terms, correlation of independent variables and goodness of fit-test for the specified model were performed.

#### Face and content validity

The meaning and acceptability of the items by the mothers were investigated by a community midwife during the administration of the scale.

#### Criterion validity

Finally, the validity of the EPDS in its Greek version - as a screening tool- was investigated considering the BDI-II diagnostic cut-off scores as a validated measure for classifying mother with depressive symptoms or with no depressive symptoms.

#### Construct Validity

Convergent validity requires that EPDS should correlate with related variables as BDI-II. Therefore, correlation coefficients (Pearsons and Spearman's rho) between global EPDS and BDI-II scores were estimated in order to determine the magnitude of the relationship between the two scales; correlation data for the two subscales -which have been revealed by factor analysis- were analysed in order to examine construct validity of the Greek EPDS.

#### Sensitivity and specificity

The sensitivity, specificity and positive and negative predictive values were calculated at several cut-off scores against BDI-II scale. A Receiver Operating Characteristic (ROC) analysis was carried out; this method allows display of all the pairs of sensitivity and specificity values achievable as the threshold is changed from low to high scores plotting the true-positive rate (sensitivity) on the vertical axis and the false - positive rate (one minus specificity) on the horizontal axis. The area under the ROC curve (AUC) is a quantitative indicator of the information content of a test and it may be interpreted as an estimate of the probability that a depressed mother chosen at random will, at each threshold, have a higher test score than a non-depressed mother.

## Results

### Sample characteristics

The response rate (99.7%) was very high. The sample demographic and obstetric characteristics are shown in Table 1. The mean age of the mothers was 29.27 years (SD = 0.489); 67 women (55.8%) were primaparae and 52 (43.3%) were multiparae. The mean EPDS score and BDI-II scores were 8.16 (SD 0.435 CI 95% 7.30-9.02) and 10.46 (SD 0.622 CI 95% 9.23-11.69) respectively. The mean scores of questions of EPDS had a range of (0.11-1.64) with question 10 and 4 to have the minimum and maximum mean score respectively.

**Table 1:** Characteristics of Sample

		**According to BDI-II**	
	**All Women** **No (%)**	**Not Depressed** **No (%)**	**Depressed** **No (%)**
**Age**			
<24	24 (20)	12 (20)	12 (20)
25-29	31 (25.8)	14 (23.3)	17 (28.3)
30-34	46 (38.3)	20 (33.3)	26 (43.3)
>35	19 (15.8)	14 (23.3)	5 (8.3)
**Education**			
Elementary & junior high	23 (19.2)	11 (18.3)	12 (20)
High School	56 (46.7)	27 (45)	29 (29)
University	33 (27.5)	16 (26.7)	17 (28.3)
Postgraduate Studies	7 (5.8)	5 (8.3)	2 (3.3)
**Work Status**			
Housewife	35 (29.2)	16 (26.7)	19 (31.7)
Unemployed	10 (8.3)	3 (5)	7 (11.7)
Student	2 (1.7)	1 (1.7)	1 (1.7)
Private Sector	29 (24.2)	16 (26.7)	13 (21.7)
Independent	13 (10.8)	6 (10)	7 (11.7)
Public Sector	21(17.5)	12 (20)	9 (15)
Other	8 (6.7)	5 (8.3)	3 (5)
**Family income per month**			
500-1000 Euros	34 (29)	15 (25)	19 (31.7)
1000 -2000 Euros	29 (24.2)	12 (20)	17 (28.3)
2000-3000 Euros	22 (18.3)	12 (20)	10 (16.7)
>3000	26 (21.7)	15 (25)	11 (18.3)
**Type of Delivery**			
Vaginal	60 (50)	29 (48.3)	31 (51.7)
Caesarean Section	60 (50)	31 (51.7)	29 (48.3)
**Gestational age of newborn**			
Preterm	10 (8.3)	6 (10)	4 (6.7)
Term	101 (84.2)	52 (86.7)	49 (81.7)
Post term	8 (6.7)	2 (3.3)	6 (10)
**Prenatal Complications**			
No	92 (76.7)	45 (75)	47 (78.3)
Yes	27 (22.5)	14 (23.3)	13 (21.7)
**Postnatal Complications for Neonatal**			
No	108 (90)	51 (85)	57 (95)
Yes	12 (10)	9 (15)	3 (5)
**Use of Drugs During Delivery**			
No	54 (45)	24 (40)	30 (50)
Yes	65 (54)	36 (60)	29 (48.3)
**Hospital/Clinic**			
Public Hospital	60 (50)	30 (50)	30 (50)
Private Clinic	60 (50)	30 (50)	30 (50)

Sixty (50%) mothers were considered to exhibit depressive symptoms on the basis of a BDI-II score more than 9; 8 (6.7%) of them were suffering from major depressive symptoms, 14 (11.7%) suffered from severe moderate depressive symptoms and 38 (31.7%) from mild depressive symptoms. The mean EPDS score was 10.42 (Std. Error 0.574 SD 4.447 CI 95% 10.994-9.846) in the depressed mothers and 5.9 (Std. Error 0.511 SD 3.956 CI 95% 4.88-6.92) in the non-depressed women. Levene's Test for equality of variances homogeneity (F = 0.781, P < 0.379) (t = -5.878 df = 118 Sig.(2-tailed) = 0.0005).

The group with depressive symptoms was divided in three subgroups (mild, moderate, severe) according to their BDI-II scores. The mean EPDS score in mothers with mild depressive symptoms (n = 38) was 8.66 (Std. Error 0.601 SD 3.707 CI 95% 7.44-9.88), in those with moderate depressive symptoms (n = 14) was 12.21 (Std. Error 0.853 SD 3.191 CI 95% 10.37-14.06) and in those with severe depression symptoms (n = 8) was 15.62 (Std. Error 1.614 SD 4.565 CI 95% 11.81-19.44) (Welch Statistic = 20.281 df1 = 3 df2 = 24.901 P < 0.0005; Brown-Forsythe Statistic = 21.721 df1 = 3 df2 = 32.737 P < 0.0005). Since the number of comparisons is larger than 5, method Tukey Statistical Significant Difference was used in the following pairs:

a. Non depressive symptoms - mild depressive symptoms, p < 0.004

b. Non depressive symptoms - moderate depressive symptoms, p < 0.0005

c. Non depressive symptoms - severe depressive symptoms, p < 0.0005

d. Mild depressive symptoms - moderate depressive symptoms, p < 0.019

e. Mild depressive symptoms - severe depressive symptoms, p < 0.0005

f. Moderate depressive symptoms - severe depressive symptoms, p = 0.192

### Psychometric characteristics of Greek EPDS

#### Reliability

The Greek EPDS showed a very high overall internal consistency (alpha value: 0.804 CI:0.108-1.642, p < 0.0001). The internal consistency characteristics of Greek EPDS showed good reliability; Cronbach's alpha was 0.804 for the total scale (Items 1-10), Standardised alpha 0.805 and Guttman split-half 0.742.

#### Factor Structure

##### Exploratory Factor analysis

**Figure 1:**
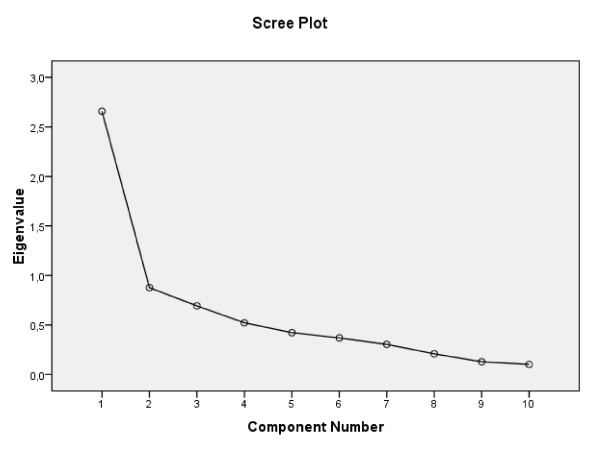
**Screeplot**.

**Figure 2:**
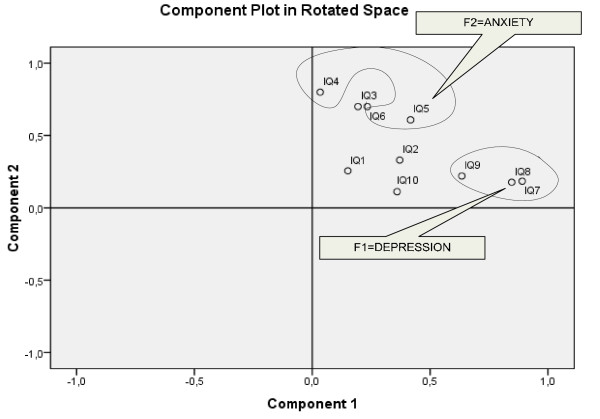
**Component Plot in Rotated Space**.

The exploratory factor analysis on the 10 items of the EPDS revealed two orthogonal factors (KMO measure of sampling adequacy = 0.787 and Bartlett's test of sphericity = 332.886, df = 45, p < 0.0005). Communalities for Greek EPDS questions are presented in Table 2. As the Screeplot (Figure 1) and Component Plot in Rotated Space (Figure 2) indicate there are two factors in the model. Those factors explained 48.97%, as presented in Table 3. The first factor (F1) includes the following items: 7 (sleep disorders), 8 (sadness) and 9 (tearfulness). These are specific symptoms for depressive disorders; therefore we named this subscale 'Depressive Symptoms'. The second factor (F2) is composed of items 4 (anxiety), 5 (panic attacks), and 6 (inability). Therefore F2 represents 'Anxiety'. The loadings of item 10 with F1 and F2 were similar.

**Table 2:** Inter- Item Correlation Matrix for Greek EPDS

	**Q1**	**Q2**	**Q3**	**Q4**	**Q5**	**Q6**	**Q7**	**Q8**	**Q9**	**Q10**
**Q1**	1.000	0.346	-0.008	0.301	0.236	0.206	0.168	0.210	0.044	0.102
**Q2**	0.346	1.000	0.273	0.293	0.321	0.255	0.293	0.484	0.246	0.199
**Q3**	-0.008	0.273	1.000	0.413	0.295	0.377	0.321	0.281	0.348	0.144
**Q4**	0.301	0.293	0.413	1.000	0.465	0.374	0.230	0.176	0.226	0.155
**Q5**	0.236	0.321	0.295	0.465	1.000	0.427	0.455	0.411	0.263	0.140
**Q6**	0.206	0.255	0.377	0.374	0.427	1.000	0.313	0.351	0.271	0.146
**Q7**	0.168	0.293	0.321	0.230	0.455	0.313	1.000	0.666	0.450	0.264
**Q8**	0.210	0.484	0.281	0.176	0.411	0.351	0.666	1.000	0.502	0.300
**Q9**	0.044	0.246	0.348	0.226	0.263	0.271	0.450	0.502	1.000	0.418
**Q10**	0.102	0.199	0.144	0.155	0.140	0.146	0.264	0.300	0.418	1.000

**Table 3:** Exploratory factors and Explained Variance after rotation for the Greek EPDS

**Factors**				**Rotation Sums of Squared Loadings**			
		
		**Rescaled** **Loadings**	**Eigen** **values**	**% of Variance**	**Cummulative** **Variance**	**Cronbach's** **alpha**	**Standardised** **alpha**
**Factor I**	**Question** **7**	0.869	2.658	27.012	27.012	0.774	0.778
						
	**Question** **8**	0.826					
						
	**Question** **9**	0.683					
						

**Factor II**	**Question** **4**	0.641	0.876	21.957	48.970	0.686	0.687
						
	**Question** **5**	0.805					
						
	**Question** **6**	0.589					

##### Confirmatory Factor Analysis

**Figure 3:**
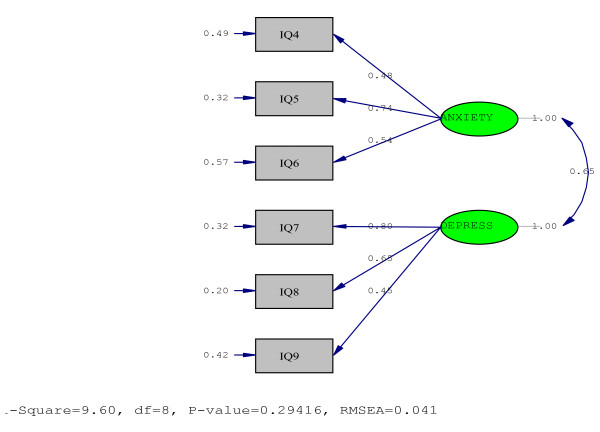
**Confirmatory Factor Analysis**.

Confirmatory factor analysis was conducted to determine whether data are consistent with the apriori specified model that has been suggested by exploratory factor analysis in order to evaluate whether the data fit the model adequately. The two factor-model was based on correlated factors that derived from the factor analysis using principal component analysis with varimax rotation by SPSS 16. The two latent variables Depress (Questions 7, 8, 9) and Anxiety (Questions 4, 5, 6) were strongly correlate (r = 0.65, p < 0.05) with method Maximum Likelihood (Figure 3). LISREL estimates, standard error, t-values, error terms and r^2 ^for all the questions that consisted each latent variables are presented at Table 4. The error terms correlated significantly (with a range of: 0.20 to 0.57) Goodness of Fit Statistics were also estimated; Minimum Fit Function Chi-Square= 9.84, p = 0.28; Comparative Fit Index (CFI) = 0.99; Goodness of Fit Index (GFI) = 0.97; Adjusted Goodness of Fit Index (AGFI) = 0.93; Standardized Root Mean Square Residual (SRMR) = 0.041.

**Table 4:** LISREL Estimates (Maximum Likelihood) for the Greek EPDS

**Independent Variables**		**Measurement Equations**				
		
		**Estimates**	**Standard error**	**t values**	**Error terms**	**R^2^**
***Latent Variable:*** ***DEPRESS***	**Question** **7**	0.80	0.085	9.45	0.32	0.66
	
	**Question** **8**	0.65	0.068	9.55	0.20	0.67
	
	**Question** **9**	0.45	0.072	6.27	0.42	0.33

***Latent Variable:*** ***ANXIETY***	**Question** **4**	0.48	0.083	5.77	0.49	0.32
	
	**Question** **5**	0.74	0.091	8.15	0.32	0.63
	
	**Question** **6**	0.54	0.091	5.91	0.57	0.34

#### Validity

##### Face and Content validity

The Greek version of EPDS was well accepted by the mothers. It was easily and very quickly (approximately 5 minutes) completed. The questions appeared to be relevant, reasonable, unambiguous and clear. Therefore, face validity was considered to be very good. The content of Greek version of EPDS includes in a balanced way the full scope of the characteristics of postnatal depression -especially anxiety and depressive symptoms- that is intended to measure.

##### Criterion validity

The overall accuracy of Greek EPDS, as a screening instrument can be described as the area under its ROC curve. The curve was plotted considering, for the EPDS scores, a range between 1 and 23 (the maximum score reached by one depressed subject in our sample). The area under the minor depression ROC curve is = 0.794 (SD = 0.048, Asymp. Sig. = 0.0005; CI = 0.700-0.888). The area under the moderate and severe depression ROC curve is = 0.902 (SD = 0.051, Asymp. Sig. = 0.0005; CI = 0.798-1.000), which is considered excellent.

Analyzing the scale sensitivity and PPV percentages in the detection of depressed women at the 8/9 cut off score the sensitivity is 76.66% specificity 68.33 and PPV is 70.76% and NPV is 74.54 (Table 5). The estimation for the threshold score of 12/13 fitted the model sensitivity at 87.5% and model specificity at 85.7%, for identifying major depression. As the threshold score increases to the cut off score of 12/13 the model sensitivity lowers while model specificity reaches higher proportions. As a result we found an optimal cut-off score of 12.5 for major depression and of 8.5 for minor, moderate and major depression.

**Table 5:** Sensitivity, specificity and positive predictive values of different cut-off scores of the Greek EPDS for identifying minor, moderate and severe depression

**Threshold** **scores**	**Sensitivity** **(%)**	**Specificity** **(%)**	**PPV** **(%)**	**NPV** **(%)**
6	88.33	51.66	64.63	81.57

7	81.66	58.33	66.21	76.08

8	76.66	68.33	70.76	74.54

9	60	78.33	73.46	66.19

10	58.33	81.66	76.08	66.21

11	48.33	86.66	78.37	62.65

12	41.66	93.33	86.20	61.53

13	33.33	95	86.95	58.76

14	23.33	95	82.35	55.33

15	16.66	95	76.92	53.27

*16*	11.66	96.66	77.77	52.25

**Figure 4:**
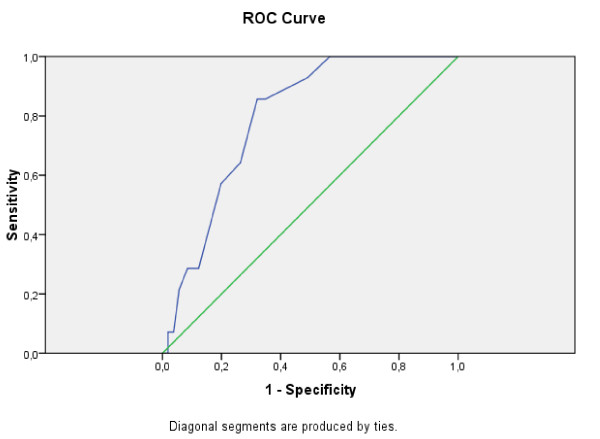
**ROC curve for Greek EPDS: Minor Depression according to BDI-II**.

**Figure 5:**
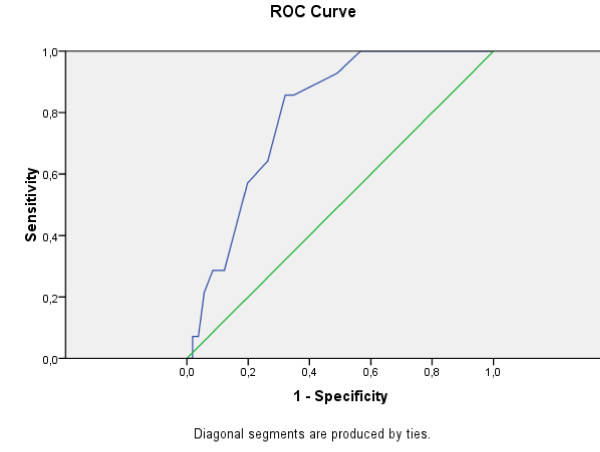
**ROC curve for Greek EPDS: Moderate and Severe Depression according to BDI-II**.

**Figure 6:**
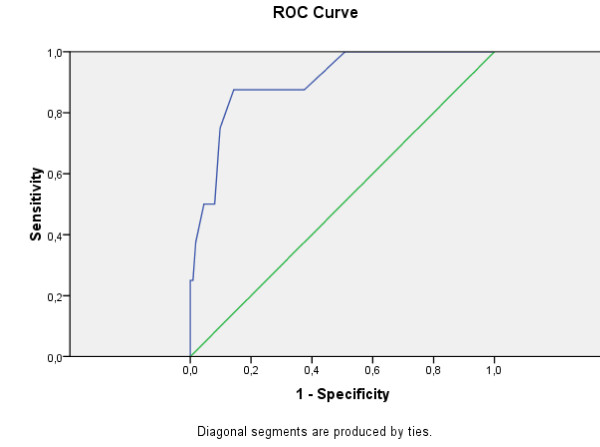
**ROC curve for Greek EPDS: Severe Depression according to BDI-II**.

Figure 4, Figure 5 and Figure 6 show the accuracy of Greek EPDS in screening the mothers that participated in this study for minor, moderate and severe depression. Using ROC Curve, we have created multiple curves in order to compare two different systems of classification, one using the cut-off score for minor depression (suitable for screening purposes) and the other using the cut-off score for major depression (suitable for diagnostic purposes) according to BDI-II. The plot of the curves offers an excellent visual comparison of the models' performances, and the area under the curve table gives evidence to back up the conclusions.

##### Construct validity

Convergent validity: the Greek EPDS (Mean = 8.16, SD = 0.435) was strongly correlated (Pearson r = 0.66 p < 0.0005) with the validated Greek version of BDI-II (Mean = 10.46, SD = 0.622) (normal distribution, linearity, homoscedacity were checked). Moreover, according to factor analysis two subscales have been revealed within EPDS. Cronbach's alpha was 0.741 for the first subscale and 0.718 for the second one.

## Discussion

EPDS is the most used scale for screening depression in postnatal period worldwide. It has already been validated in many countries such as The Netherlands [25], Portugal [26], Sweden [27], and Australia [28] and has shown remarkable stability and comparability.

Cronbach standardised alpha and Guttman Split-half for the Greek EPDS were found similar to those reported by Cox in the first validation study (0.87) [4], by Pop et al in the Dutch validation study (0.82) [25] and Benvenuti et al in the Italian validation study (0.78) [29]. The mean EPDS score in the depressed women was 10.42 and the non-depressed women 5.90 (F = 0.781, P = 0.379) (t = -5.878 df = 118 Sig.2-tailed = 0.0005), while in the Italian validation study reported mean EPDS score in the depressed women 13.6 and for the non-depressed women 5.1 [29], in the Swedish validation study reported higher mean EPDS scores (15.4 for the depressed women and 10.4 for the non-depressed) [27].

A limitation of this validation study was that there was no test-retest, because it may have resulted in a low correlation due to an actual change in the depressive symptomatology. More over, the depressive symptomatology was assessed with only two paper-and pencil measures (i.e. EPDS and BDI-II) without further evaluation through clinical interviews which may have resulted in diagnosis or treatment of clinical postnatal depression. Despite the above limitation, -as in other previous international studies [5] - this study investigates the association between the two widely used depression measures (EPDS and BDI-II) by comparing their scores also in a Greek sample of mothers. Regardless of the small targeted population and sample size, participants were representative of the populations (urban and rural) served by the four recruiting hospitals. Rapid socioeconomical changes over the last three decades, have led to a relatively homogenous cultural background of cretans with the rest of Greece. In spite of the above concerns, the size of our sample is considered excellent for explanatory and confirmatory factor analysis.

Since Cox et al suggested that EPDS has one dimensional aspect [4], a number of studies that have examined its structure, have found the EPDS to be multidimensional and that it can be distinguish at two factors; however, significant variation has been observed between the item factor loadings between studies [25,30-36]. The two sub-scales of Greek EPDS showed very good alpha values, similar to those found by Pop et al [25]. Our findings confirm the multidimensionality of EPDS, demonstrating a two-factor structure with similar loadings, while recent studies have demonstrated postnatal significant differences in item-factor loadings characteristics [30-34,36]. These findings may be explained by the different periods of application of EPDS or the different culture backgrounds. The Confirmatory Factor analysis demonstrated that the two factor model based on the Explanatory Factor Analysis offered a very good fit to the our data, in comparison to other two and three factor models that have been introduced by other researchers [30-34,36]. All Goodness of Fit Statistics found to be very good since they are all approaching 1. Especially SRMR(= 0.041) is excellent, since it has a range of 0 to 1 and values of 0.08 or less are desired [37].

It has been argued that factor stability is important for the explanatory value of a predictor sub-scale, as it demonstrates the ability to be explained in the criterion or target variable [30-32]. However, it is important not to underestimate the social and clinical significance of item 10. This item should be regarded as essential to the content validity of the measure, though it doesn't load on a cluster of inter-related variables, its retention as separate item in EPDS scale should be considered on theoretical grounds [17].

Although the first validation study [4] suggested the 9/10 cut off score for the use of the scale in the community surveys and screening, the 12/13 threshold was more useful in the clinic assessment of the postnatal depression. A community sample of randomly selected postpartum women was screened and found lower sensitivity value and positive predictive value: 67.7% and 66.7% respectively [38].

A threshold of 11/12 was reported as more suitable for screening a French population [39]; a sensitivity of 96%, a specificity of 49% and PPV of 59%, using cut- off of 11/12 was reported for the Swedish population [27]; a cut-off score of 8/9 (sensitivity 94.4%, specificity 87.4% and PPV 58.6%) was more appropriate in an Italian population [29], a cut-off score of 9/10 was appropriate for screening Chinese population (sensitivity 82%, specificity 86% PPV 44%) [40] and a cut-score of 9 was appropriate for Japanese population, giving a sensitivity of 75% and a specificity of 93% [41].

The ROC analysis confirmed the effectiveness of EPDS in detection of postnatal depression as well as its application in the range of cut-off scores proposed in previous studies. In our study, the high sensitivity (76.66) associated with a good PPV (70.76) to the 8/9 cut-off score allows the use of this score in the community screenings. Our choice of cut-off score has been mandated by the need to screen mothers to prevent postnatal depression rather than for diagnostic purposes. It is worthwhile to note that these cut-off values are at best guidelines for which cut-offs a health professional should consider for screening purposes. If a health professional would like to use the Greek EPDS for diagnosis, then different cut-offs -based on major depression scores according to BDI-II- should be used. Additionally, ROC analysis does not provide error estimates, so there is no guarantee of the accuracy of the sensitivity or specificity for a given cut-off.

Moreover, the prevalence rate for major postnatal depression of 6.7% in this study is consistent with reported rates in the literature [2]. This similar prevalence rate is important to the psychometric testing of Greek EPDS as a screening instrument, as predictive values are very much influenced by the prevalence of postnatal depression [42]. As a result, the screening instrument will have decreased positive predictive value and increased negative predictive value in clinical practice. The implication for practice is thus a low probability of being depressed, if a mother has a positive EPDS screening. However, efforts were made to recruit a representative sample from the specified geographical area. Since the results of this study show a considerable similitude with those found in the previous validation studies, in particular with the prevalence of postnatal depression in the sample, similar predictive values for EPDS as a screening tool would be obtained if used in clinical practice. It is very important for the EPDS to be used as screening scale in clinical practice, as routine screening of mothers may allow the practicing midwife to facilitate an accepting dialogue with mothers with a devastating mood disorder.

## Conclusion

The Greek version of the EPDS has shown a satisfactory reliability and factor analysis indicated by two components similar to those of the original version. ROC analysis versus BDI-II provides the cut-off score of 8.5 as the best one for screening mother for minor, moderate and severe depression. We can therefore assert that it is a reliable and valid tool for identifying postnatal depression and it can be used by health professionals in their clinical practice to improve early detection, assessment and treatment for mothers with high scores.

## Abbreviations

EPDS: Edinburgh Postnatal Depression Scale; BDI-II: Beck Depression Inventory- II; ANOVA: One way analysis of variance; LISREL: Linear Structural Relations; ROC: Receiver Operating Characteristic; PPV: Positive Predictive Value; NPV: Negative Predictive Value; AUC: Area Under Curve; KMO: Kaiser-Meyer-Olkin.

## Competing interests

The authors declare that they have no competing interests.

## Authors' contributions

VV participated in study design, translation, adaptation and validation of the questionnaire, carried out data collection and data entry, participated in the analysis and wrote the final draft of the manuscript. VD participated in study design, carried out the statistical analysis and co-wrote the final draft of the manuscript. MK and PB provided consultation during translation/adaptation/validation process and commented on the writing of the final draft of the manuscript. CL conceived the study design, was coordinator in the translation/adaptation/validation process and co-wrote the final draft of the manuscript. All authors read and approved the final manuscript.

## Pre-publication history

The pre-publication history for this paper can be accessed here:


